# Authors’ Reply: Who Reviews the Rewrite? Separating Patient-Initiated and Institutionally Curated Large Language Model Simplification

**DOI:** 10.2196/107588

**Published:** 2026-08-31

**Authors:** Amela Miftaroski, Richard Zowalla, Martin Wiesner, Monika Pobiruchin

**Affiliations:** 1 Faculty of Informatics, Heilbronn University, Max-Planck-Str. 39, Heilbronn, Baden-Wurttemberg, 74031, Germany, 49 7131504 ext 633; 2 Research and Innovation Center for Cognitive Service Systems (KODIS), Fraunhofer Institute for Industrial Engineering, Stuttgart, Germany; 3 Consumer Health Informatics Special Interest Group, German Association for Medical Informatics, Biometry and Epidemiology (GMDS) e.V, Cologne, Germany

**Keywords:** health information, patient education material, readability, large language models, LLMs, AI

We thank the correspondent [1] for the close, constructive reading of our study [2]. The letter’s most valuable contribution is its framing: patient-initiated and institutionally curated large language model (LLM) simplification are distinct workflows, and future studies should prespecify which they are evaluating. We welcome this organizing principle and will adopt it in future work. For accuracy, we note that we already distinguish these perspectives and do not conflate them. Our design reflected the layperson’s vantage point, and our Innovation section explicitly turns to the other: “[s]hifting from the perspective of citizens and patients to health professionals or institutions: The use of an LLM could be a time-saving and cost-effective tool to fine-tune their information leaflets, online texts, etc to meet different information needs.” What the letter adds is the requirement that the workflow be prespecified as a design parameter, sharpening a distinction the paper (already) drew.

We agree with the letter’s closing formulation—that medical text may become easier to read without becoming safer to understand. This was our own published conclusion: the use of LLMs “requires careful expert review to ensure accuracy and completeness of medical information”; “[s]ome rephrased texts conveyed incorrect messages or took statements out of context. This is a serious risk, especially for medical texts”; and “a manual check is still needed and advised when using LLMs in similar scenarios.” We are pleased that an independent reader has made the same conclusion, and we take this as confirmation rather than correction.

Three points warrant clarification: first, the correspondent assumes “that the panel had heterogeneous academic backgrounds.” In fact, the paper mentions that “3 reviewers (AM, RZ, MP) [had] a background in medical informatics,” that is, a shared background, and we identified the absence of deep clinical expertise as a limitation. Second, we explicitly characterized the secondary driver of low agreement: “the difficulty of evaluating medical content without deep domain-level expertise,” noting that reliability was “further complicated by uncertainty regarding the correctness of the original websites.” Third, we didn’t infer that unreviewed LLM simplification improves patient understanding. Our conclusions are confined to *readability*, qualified as moderate, and conditioned throughout on expert review.

The scope of the accuracy component follows directly. We aimed to investigate, from a layperson’s perspective, whether LLMs can increase the readability of German online medical texts, reaching the recommended eighth-grade level. Systematic evaluation of medical accuracy was expressly placed outside that aim in our Limitations: “[t]he systematic evaluation of the (medical) accuracy of rephrased PEMs [patient education materials] was beyond the study’s scope.” The accuracy aspect was described in the Results as “not a systematic assessment.” A nonsystematic secondary screening cannot reasonably be faulted for lacking the apparatus of a systematic one. The Guidelines for Reporting Reliability and Agreement Studies [3] address studies that investigate reliability as an outcome; the guidelines prescribe disclosure rather than calibration, rubrics, or adjudication, and we do not think they are the governing standard for an exploratory step of this kind.

Nevertheless, we accept that we did not publish a prespecified rating rubric, did not report reviewer-level ratings or an adjudication procedure, and did not state whether accuracy, clarity, and plausibility were scored as separate constructs or a single judgment. These are details for a purpose-designed accuracy study. They do not disturb any published finding, because the qualitative observations are existence claims that remain individually verifiable against their source texts—the Copilot compression of the endometrial cancer material and the decontextualized myocarditis statement. They are documented in the paper and stand or fall on inspection, not on the achieved κ.

On construct validity, we agree entirely, and we never claimed otherwise. As the paper states, “[b]oth metrics use text parameters like average sentence length and average number of syllables per word”; the Flesch Reading Ease in Amstad’s German recalibration and the Wiener Sachtextformel quantify linguistic features and do not establish whether a reader is informed or can act. We chose them for comparability with prior German-language patient education material research [4], and our Limitations section acknowledged that “aesthetic and design features or educational multimedia can influence the *understandability* of information material.” The Patient Education Materials Assessment Tool (PEMAT) [5] and direct comprehension testing are sound complements, and we endorse them as future directions, although PEMAT is not validated for German and would require adaptation and revalidation before use.

In closing, we agree with the correspondent that readability is necessary but not sufficient for safe patient communication; the patient-initiated versus institutionally curated distinction is a good organizing principle, and the open question—whether LLM output combined with expert review measurably outperforms conventional human authorship—is the right one to ask next.

## Abbreviations

LLM: large language model
PEMAT: Patient Education Materials Assessment Tool
